# A Comparison of the Electronic Properties of Selected Antioxidants Vitamin C, Uric Acid, NAC and Melatonin with Guanosine Derivatives: A Theoretical Study

**DOI:** 10.3390/molecules29245944

**Published:** 2024-12-17

**Authors:** Boleslaw T. Karwowski

**Affiliations:** DNA Damage Laboratory of the Food Science Department, Faculty of Pharmacy, Medical University of Lodz, ul. Muszynskiego 1, 90-151 Lodz, Poland; boleslaw.karwowski@umed.lodz.pl

**Keywords:** antioxidants, guanine derivatives, ionisation potential, electron affinity, second-or-der Møller-Plesset perturbation theory

## Abstract

Each cell in the human body is continually exposed to harmful external and internal factors. During evolution, cells have developed various defence systems, divided into enzymatic and non-enzymatic types, to which low-weight molecule antioxidants belong. In this article, the ionisation potential and electron affinity, as well as global reactivity descriptors of Vitamin C, Melatonin, Uric Acids, and N-acetyl-L-cysteine, were theoretically investigated at the MP-2/aug-cc-pVTZ level of theory in the condensed (aqueous) phase. The vertical ionisation potential and electron affinity are discussed in terms of non-equilibrated and equilibrated solvent–solute interactions. Additionally, at the same theoretical level, the electronic properties of canonical and oxidised derivatives of guanine were analysed. The presented results indicate that the selected antioxidants for this study (Vitamin C, Uric Acid, NAC, and Melatonin) exhibit the highest adiabatic electron affinity, while guanine derivatives (Gua, ^OXO^Gua, Guo, dGuo, ^OXO^Guo, ^OXO^dGuo) are more prone to adiabatic radical cation formation. A red-ox balance (redox homeostasis) is crucial for intracellular signalling pathways that are reactive oxygen and nitrogen species (RO/NS)-dependent. Should this gentle balance be disrupted, either by an overload or deficit of species, physiological consequences may result, which in turn lead to pathological outcomes. On the other hand, maintaining the stability of the above balance of antioxidants/radicals may result in the improved effectiveness and safety of anticancer radiotherapy/chemotherapy or combined therapies with a subsequent increase in a patient’s quality of life.

## 1. Introduction

The human body contains around 10^14^ cells, which mutually interact and communicate through intracellular media [1,2]. A similar number of physiological bacterial cells are found within the human microbiome. They are all continuously exposed to harmful external physicochemical factors such as radiation, xenobiotics, temperature, UV, pharmaceutical drugs, reactive oxygen and nitrogen species (RO/NS), etc. [3]. On the other hand, intracellular processes lead to the formation of reactive molecules such as metabolic and lipid peroxidation products, RO/NS, and solvated electrons [4]. Their main sources of reactive oxygen species in a cell are the endoplasmatic reticulum (microsomal oxidation), lysozymes (myeloperoxidase), mitochondria (electron transport), peroxisomes (oxidases), and xanthine [5]. These factors can lead to injury of the lipid bilayer, the destabilisation of genetic information, the inactivation of enzymes and other proteins, or a change in their function [6]. As a result, during evolution, cells have developed systems to protect against highly reactive species such as radicals [7]. These defence systems are either enzymatic or non-enzymatic. The main proteins involved in the inactivation of reactive oxygen species are catalase, superoxide dismutase, and glutathione peroxidase [8].

**Figure 1:**
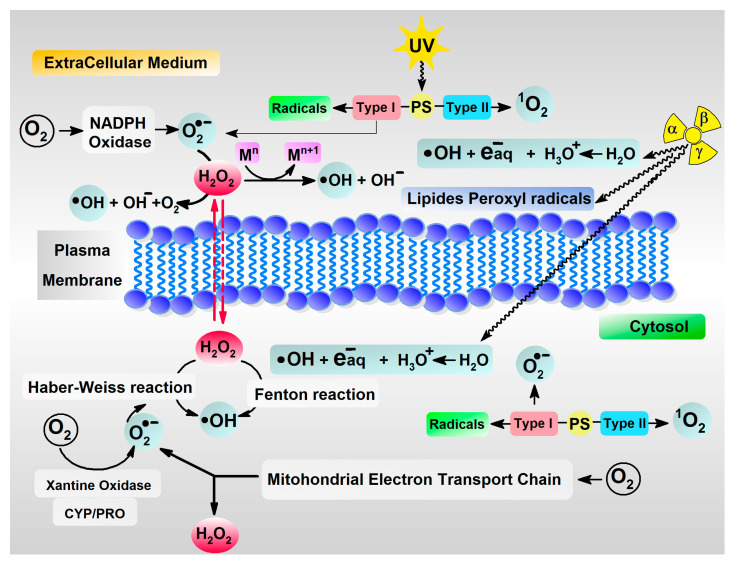
The extracellular and endocellular sources of free radicals and reactive oxygen species with selected factors indicated, i.e., α, β, γ ionisation radiation, exposed to UV, activity of photosensitiser (PS) type I and II, endogenous metabolic reactions catalysed by various proteins and transient metals ions.

The most abundant and vital element for life, oxygen, can be converted into reactive species such as H_2_O_2_ (hydrogen peroxide), O_2_^•−^_n_(superoxide anion radical), •OH (hydroxyl radical), and ^1^O_2_ (singlet oxygen), which are potential threats to living cells if not inactivated [9]. As shown in Figure 1, ROS can be formed during the activity of various internal and external factors. It should be highlighted that reactive oxygen species can be present on both sides of the cellular membrane, i.e., in the intercellular space and in the cytosol itself. Disruption of this delicate balance as a result of infection can lead to various pathological processes including carcinogenesis, diabetes, and neurodegenerative disorders [10]. On the other hand, macrophages and neutrophils utilise HOCl for pathogen inactivation by the generation of oxidative stress [11]. Myeloperoxidase catalyses the formation of HOCl from H_2_O_2_ [12], which plays a crucial role in blood vessels. The endothelial layer is susceptible to ROS, particularly in pathological states [13]. During the breathing process (gas exchange) when the vital element of oxygen is inhaled, air pollutants (e.g., from burn and smoke inhalation [14]) are also introduced, increasing the exposure of lung cells to inflammatory processes.

Non-enzymatic antioxidants in the human body when consumed with food can directly support the defence system after the exhaustion of the enzymatic antioxidant system or in response to RO/NS accumulation, overload, or overproduction. Their radical neutralisation properties depend on the molecule’s capability for electron loss or adoption. In light of the above, this study considers the electronic properties of common low-molecular-weight antioxidants and compares them with guanine derivatives present in the cytosol and extracellular fluids.

## 2. Results

**Figure 2:**
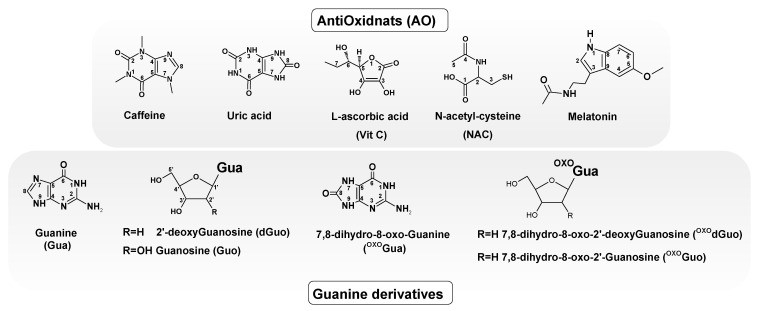
Graphical representation of selected antioxidants (Vit C, Melatonin, NAC, and Uric Acid) and canonical derivatives (Gua, Guo, dGuo) and post-oxidation guanine derivatives (^OXO^Gua, ^OXO^Guo, ^OXO^dGuo) with suitable atom numbering.

The geometry optimisation of the selected antioxidants Vitamin C (Vit C), N-acetyl-cysteine (NAC), Melatonin, Uric Acid, Melatonin and guanine derivatives, i.e., guanine (Gua), 2′-deoxyguanosine (dGuo), guanosine (Guo), 7,8-dihydro-8-oxo-2′-deoxyguanosine ^OXO^dGuo), and 7,8-dihydro-8-oxo-guanosine (^OXO^Guo), was performed at the MP-2/aug-cc-pVTZ level of theory in the aqueous phase using the conductor-like polarisable continuum model (CPCM) [15]. Figure 2 presents the structure and suitable atom numbering of the discussed molecules. Because water is the medium for all biochemical reactions and becomes at least 65% of the human body/cell, all of the theoretical experiments were conducted in the aqueous phase [16]. It should be highlighted that because H_2_O particles interact randomly with molecules, the CPCM model was used for this study. (The variations in the results may arise from the presence or absence of a hydrogen bond interaction and the arrangement of aqueous surfaces inside the microsolvation model or periodic box). Antioxidants have been defined as “any substance which can delay or prevent the oxidation of a substrate when it is present in small amounts relative to the amount of the substrate” [17]. According to Halliwell, these molecules can (a) decrease localised oxygen concentrations, (b) prevent chain initiation by scavenging initiating radicals, (c) bind catalysts such as metal ions to prevent radical formation, (d) decompose peroxides, and (e) disrupt the chain reaction thus preventing continued hydrogen abstraction by active radicals [18].

### 2.1. Electronic Properties

**Figure 3:**
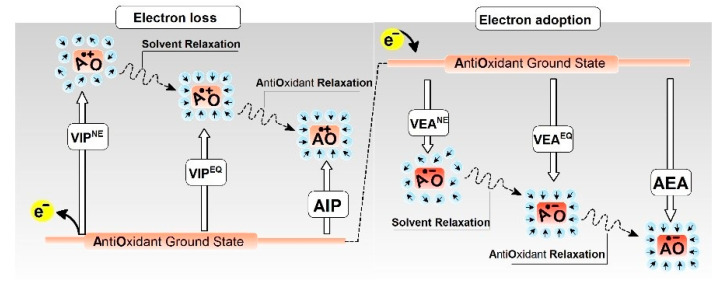
Schematic representation of radical cation (electron loss) or radical anion (electron adoption) movement during geometry relaxation and electronic parameters of this process with reference to the state of the solvation environment. VIP^NE^—vertical ionisation potential in non-equilibrated (NE) solvent mode, VIP^EQ^—VIP in an equilibrated (EQ) solvent state, AIP-adiabatic ionisation potential, VEA^NE^—vertical electron affinity in non-equilibrated solvent mode, VEA^EQ^—VEA in an equilibrated solvent state, AEA-adiabatic electron affinity: e^−^—electron, AO—antioxidant. The details have been given in the Materials and Methods section with a mathematical description.

The properties of the discussed molecules predict their ability to lose or adopt electrons, i.e., their ionisation potential or electron affinity. According to Koopmans’ theorem, the HOMO (highest occupied molecular orbital) energy of a molecule can be regarded as the vertical ionisation potential, while the LUMO (lowest unoccupied molecular orbital) energy can be considered the vertical electron affinity [19]. It should be noted that the obtained results describe the energy necessary for an electron to be removed from or settle onto the valence orbital. In the above model, the changes in solvent–solute interaction were also considered in this study. Using Koopmans’ theorem, the lowest HOMO energy among all the discussed antioxidants was found for Melatonin, at 8.38 eV, which is similar to the values assigned for the discussed Gua derivatives (Table 1). (The exception is ^OXO^Gua, which was lower by 0.16 eV). As for the LUMO energies, the lowest value found was close to 0.78 eV for Caffeine and Vit C (Table 1). The above results indicate that following Koopmans’ theorem, among the discussed antioxidants, Melatonin demonstrates the highest ability for electron loss (a lower ionisation potential). Conversely, Vit C and Caffeine are predisposed to electron attachment (a higher electron affinity). It should be highlighted that a solvated electron can, for example, appear during water radiolysis and change the oxidation state of cofactors, surface polarisation, etc. To discuss the electronic properties of biomolecules, it is necessary to consider the effects of the solvent shell. Therefore, the electron distribution on the molecular orbitals in both non-equilibrated (NE) and equilibrated (EQ) solvent–solute interaction modes have been considered (Figure 3) [20].

### 2.2. Ionisation Potential

For molecules in a neutral ground state, electron loss leads to the formation of a radical cation (i.e., a hole), while the attachment of an extra electron results in the formation of a radical anion. Therefore, in the initial step of electron loss or adoption, without solvent reorganisation, the vertical (V) value of the ionisation potential (IP) or electron affinity (EA) in the NE mode should be assessed. (In this stage, the vertical radical cation/anion is surrounded by a nonrelaxed solvation shell). As shown in Table 1, the following order of VIP^NE^ was observed: NAC > Vit C > Caffeine > Uric Acid > Melatonin. Moreover, the VIP^NE^ found for Melatonin was lower by 0.25 eV than all the discussed guanine derivatives. The subsequent solvent reorganisation to the equilibrated mode causes the VIP^EQ^ to decrease in a range between 0.6 and 1.4 eV (in this stage, the solvent shell was relaxed, while the molecule geometry after electron loss/adoption was still in vertical mode). Furthermore, solvent rearrangement leads to different VIP^EQ^ orders than previously: Vit C > NAC > Melatonin > Uric Acid > Caffeine. For the Gua derivatives, the VIP^EQ^ was between 6.44 and 6.96 eV (Table 1). The geometry relaxation gives rise to an adiabatic state with the following order of adiabatic ionisation potential (AIP) observed for the antioxidants: NAC > Vit C > Melatonin > Caffeine > Uric Acid. Subsequently, the adiabatic ionisation potential (AIP) of ^OXO^Gua, ^OXO^dGuo, and ^OXO^Guo decreases to as little as 5.92 eV, which is lower than the values obtained for the remaining antioxidants. For canonical Gua, dGuo, and Guo, the assigned AIP was found to be close to 6.39 eV (the average value of canonical forms).

### 2.3. Electron Affinity

The ability of molecules to adopt an extra electron and radical anion formation are described by electron affinity (EA) parameters, as shown in (Figure 3). The results presented in Table 1 reveal that in the non-equilibrated solvent–solute interaction state, the electron attachment process is unfavourable. In each case, negative VEA^NE^ values were found. However, the values calculated for Caffeine and Vit C were found to be close to 0 eV. The adoption of an extra electron by the molecule and the subsequent solvent relaxation leads to vertical radical anion formation in an equilibrated state for which the following order of VEA^EQ^ was noted: Vit C > Uric Acid > Caffeine > NAC > Melatonin. For the guanine derivatives, the order was ^OXO^Guo > ^OXO^dGuo > ^OXO^Gua > Gua > dGuo > Guo. With further geometry relaxation of the vertical radical anions, the AEA values were as follows: Vit C > Uric Acid > NAC > Caffeine > Melatonin. For the Gua derivatives, the order of AEA values was the same as established for VEA^EQ^.

### 2.4. Global Reactivity Descriptors

The energy gap between the HOMO and LUMO (Δ*E*^H-L^ = *E^HOMO^* − *E^LUMO^*) is related to the compound stability and chemical reactivity [21,22]. Hence, the greater the energy gap between the valence orbitals, the higher the molecule’s stability. As presented in Table 1, melatonin exhibited the smallest HOMO–LUMO gap, so it can be surmised that it is the most chemically reactive among all the discussed molecules. A higher (9.30 eV) Δ*E*^H-L^ was noted for NAC, while all the Gua derivatives exhibited similar values in the range of 7.25 to 7.53 eV. The above observations are in good agreement with previously calculated vertical ionisation potentials. Based on Koopmans’ theorem for closed-shell molecules, the following parameters can be calculated (in addition to VIP and VEA): the chemical hardness (η = 0.5(VEA − VIP) and softness (*S* = 1/η), the global electrophilicity index (ω = *μ^2^/2η*), and the electronic chemical potential (*μ* = 0.5(VEA + VIP). The following parameters were also calculated for the different molecular states (vertical NE/EQ and adiabatic) to show the changes in the above properties during the solvent and solute relaxation processes [23]. The results are presented in Table 2. Among the antioxidants under discussion, NAC (4.65 eV) exhibited the highest η value, which predicts its stability, while Melatonin (3.39 eV) had the lowest value and is, therefore, more reactive than the others. According to Pérez, molecules can be regarded as strong electrophiles when the global electrophilicity index (ω) is above 1.5 eV, weak when it is below 0.8 eV, and moderate for ω values between 1.5 and 0.8 eV [24]. The results of the presented studies show that all the discussed molecules are strong electrophiles (Table 2) regardless of the method used to calculate the ionisation potentials and electron affinities. The electronic chemical potential (μ) indicates the tendency of the investigated system to lose an electron or adopt an electron. A high negative μ value suggests that the molecule properties are good electron acceptors. Conversely, a lower negative μ value suggests a predisposition for electron donation.

In these studies, NAC exhibited the highest negative *μ* (−5.47), while Melatonin had the lowest (−4.33 eV). The above is in good agreement with the results obtained for VIP and VEA according to Koopmans’ theorem (Table 2). Moreover, all the guanine derivatives exhibited *μ* values close to −4.60 eV. A comparison of the chemical hardness and softness of the antioxidants and Gua derivatives showed that the antioxidants had a higher hardness and a lower softness. Moreover, the utilisation of vertical non-equilibrate, vertical equilibrate, and adiabatic states for the computation of the η, S, *μ*, and ω parameters elucidated in each case decreases in chemical hardness together with system relaxation accompanied by increases in softness. Additionally, both molecule systems, i.e., the antioxidants and Gua derivatives, exhibited a decrease in η with a subsequent rise in ω in the following order of states: Vertical^NE^ → Vertical^EQ^ → Adiabatic (Table 2).

### 2.5. Spin Distribution of the Radical Cation and Radical Anion

The spin distribution was calculated at the MP-2/aug-cc-pPVTZ level of theory in the condensed phase using Hirshfeld methodology. As expected, it was observed that the spin density reorganised during changes in solvent–solute interactions for non-equilibrated, equilibrated, and adiabatic states. For the series of Gua derivatives, the highest concentrations were found at C5 and O6 for ^OXO^Gua, ^OXO^dGuo, and ^OXO^Guo, while in the case of canonical Gua, dGuo, and Guo, the highest concentrations were at C5 and C8 (Table 3). The appearance of an extra electron in the discussed system led to initial accumulations at the N2 and N1 heteroatoms (except for Gua, which was at N9). During subsequent solvent relaxation, higher spin densities were found at the C4 and C6 carbons in the case of the ^OXO^Gua series. For the canonical Gua derivatives, the unpaired electron settled on C4 and C6 and after ribose or 2-deoxyribose attachment on C6 and C4. For the adiabatic state, the highest spin accumulation was noted at the C4 and C6 atoms of the ^OXO^Gua series and at C6 and O6 for the Gua derivatives. For Caffeine, NAC, Vit C, Melatonin and Uric Acid, in almost all the cases, the unpaired electron delocalised within the molecule matched the changes in the solvent–solute interactions after electron loss or adoption. For all the radical cation forms, only in the case of NAC was the unpaired electron located on the sulphur atom. In conclusion, the comparison of the results presented above indicates the significant role of the environment on the spin distribution within molecules, which alters their chemical properties, as presented in Table 2.

## 3. Discussion

ROS or NOS can appear in the cell as sources of the activity of various factors, as incomplete oxygen deactivation by a four-electron reduction process results in water molecules, i.e., O_2_ → O_2_^•−^ → H_2_O_2_ → •OH + OH^−^ → 2H_2_O. Reactive oxygen species can play a significant role depending on their intracellular and extracellular environment, concentration, time, and compartmentalisation. However, their half-lives are short, as follows in seconds [s]: (•OH) 10^−10^ s, (O_2_^•−^) 10^−6^ s, (ROOH, H_2_O_2_) stable, (^1^O_2_) 10^−6^, (HOCl and HOBr) a few minutes, (ONOO^−^ peroxynitrite) 10^−3^ s [25]. Of these, the hydroxyl radical is the most reactive with a reaction rate constant in biological fluids at a level of 10^9^–10^10^ M^−1^ s^−1^ and cannot be removed enzymatically, unlike ONOO^−^ [26,27]. Furthermore, their reactive space is diffusion-limited to approximately 50 molecular diameters from the site of formation. The oxidation–reduction potential of •OH has been assigned at the level of *E*_0_ (H_2_O/•OH) = 2.32V at pH = 7 [28]. Therefore, a highly efficient protective system is required. One component of this system comprises enzymes such as SOD (superoxide dismutase), catalase, and glutathione peroxides, which act as guardians against ROS production on both cell membrane sides. The second component comprises low-weight antioxidants, which effectively quench ROS that have escaped during the respiratory cycle or as a result of the activity of various factors (Figure 1). Their activity depends on their concentration in the cytoplasm and extracellular fluids. The following plasma concentrations have been assigned: Vit C: 50–200 μM [29], Melatonin: 40–100 pg/mL [30] Caffeine: 52 μM [31], NAC: 141.5 μM/L [32] and Uric Acid: 150–450 μM [33]. It should be highlighted that nucleosides are an abundant part of the cytosol before their incorporation into DNA or RNA as well as their conversion to ATP, cAMP, etc.: ATP: 3152, GTP: 468, UTP: 567, CTP: 278; and for 2′-deoxynucleotides: dATP: 24, dGTP: 5.2, dCTP: 29, dTTP: 37 [34]. The lowest ionisation potential among all the canonical nucleobases has been observed for guanine (Gua) and its nucleoside derivatives dGuo, i.e., 8.1 ± 0.2 eV [35] and 8.6 eV [36], respectively (in the gaseous phase). Due to the above, they are able to neutralise unwanted reactive molecules. Moreover, the oxidised guanosine derivatives, i.e., ^OXO^Gua, ^OXO^dGuo, and ^OXO^Guo, exhibit a lower ionisation potential than the antioxidants under discussion (Table 1), supporting their protective role in cells. Furthermore, Uric Acid is the product of guanine and adenine deamination with subsequent oxidation by xanthine oxidase, which makes it an abundant molecule in plasma. As shown in Table 1, Uric Acid exhibits the lowest AIP (6.21 eV), the lowest chemical hardness (2.32 eV) and the highest chemical softness (0.43 eV) among all the examined antioxidants. Moreover, the calculated AIP adopted a value between those assigned for Gua and ^OXO^Gua, i.e., 6.42 eV and 5.94 eV, respectively. In this study, the following order of AIP was found for the discussed antioxidants: NAC > Vit C > Melatonin > Caffeine >Uric Acid and AEA Vit C > Uric Acid > NAC > Caffeine > Melatonin. The parameters discussed above, together with the chemical properties such as hardness (η) and softness (S), justify the selection of these molecules as free radical scavengers. It should be highlighted that Melatonin is a small hormone (a metabolic product of serotonin) present in the brain that helps with the timing of circadian rhythms and sleep. Its presence in the central nervous system can protect neurons and other cells, such as astrocytes, against RO/NS activity. This is important because the delivery of many compounds through the blood–brain barrier is prohibited and does not occur. The second of the discussed antioxidants, *N*-Acetylcysteine, serves two roles: a direct antioxidant and the substrate for reduced glutathione (GSH) biosynthesis [37]. The level of glutathione in most cells has been assigned as 1–2 mM, 10 mM in hepatocytes, and 4.4–9.1 μM in plasma [37,38,39]. Significantly, the spin is always concentrated on the sulphur atom regardless of the NAC radical cation state (Table 3). After radical deactivation, GSH is converted into an oxidised dimeric form (GSSG), which is regenerated by NADPH. On the other hand, GSH can prolong the stability of Vit C as an antioxidant and protect cells from its negative effects [40,41]. Vitamin C is one of the most common and important antioxidants that exhibits a relatively moderate AIP, i.e., 6.82 eV, while its electron affinity among all the molecules discussed here was observed as the highest (Table 1), i.e., 1.62 eV. This predisposes it towards solvated electron neutralisation or other radical anions. A solvated electron is the product of processes such as water radiolysis. The final antioxidant taken into consideration in this study is Caffeine. This alkaloid is commonly present in the human diet and is found in high amounts in coffee, chocolate, and tea. Given this, its abundance in human body fluids and cells is hardly surprising. Caffeine’s hardness and softness were found as follows: 3.93 eV and 0.25 eV, respectively, which is similar to the values assigned for Melatonin (Table 2). Moreover, the calculated AIP of Caffeine was similar to Melatonin with a subsequently higher AEA, i.e., 1.10 eV and 0.68 eV respectively. The small value of the Δ*E^H-L^* parameter noted for Caffeine and Melatonin, i.e., 6.77 eV and 7.86 eV, renders these antioxidants effective radical scavengers (the lower the Δ*E^H-L^*, the higher the reactivity) (Table 1).

## 4. Materials and Methods

### Details of Theoretical Calculation

The initial geometries of the discussed molecules were built using GaussView 5 software [42]. A graphical representation is shown in Figure 2. The second-order Møller–Plesset perturbation theory (MP-2) was used for all the calculations due to its accuracy, efficiency, and reasonable calculation time [43].

The structure optimisations of all the molecules were performed at the MP-2/aug-cc-pVTZ level of theory (using the following basis set for heavy atoms and hydrogen: 5s4p3d2f and 4s3p2d, respectively) in the aqueous phase [44,45]. The same level of theory was used for all the energy calculations. Each theoretical experiment was performed in the condensed phase using Tomasi’s polarised conductor-like continuum model (CPCM) with a water dielectric constant ε = 78.4. For all the optimised geometries, a charge and spin analysis was achieved using Hirshfeld methodology at the MP-2/aug-cc-pVTZ level of theory in the condensed phase [46]. The electronic properties of the molecules were calculated at the above level of theory. The solvent effect was analysed in two modes following the previously described methodology, i.e., the non-equilibrium (NE) and equilibrated (EQ) conductor-like polarisable continuum models [47,48]. The energy of the molecule in the non-equilibrated solvent–solute interaction mode was calculated using two-step processes according to save-read procedures [47].

The following energy notation, as presented in Figure 3, was used: the *E*_geometry_^charge^ of the molecule (neutral form, ground state) is described as *E*_0_^0^, the vertical cation/anion in the non-equilibrated solvent–solute interaction mode as *E*_0_^+(NE)^/*E*_0_^−(NE)^, the vertical cation/anion in the equilibrated solvent–solute interaction mode as *E*_0_^+(EQ)^/*E*_0_^−(EQ)^, and the adiabatic cation/anion as *E*_+_^+^/^+^*E*_−_^−^.

The difference, given in eV, between the mentioned energies corresponds to the relevant electronic states graphically presented in Figure 3 and described as follows: VIP^NE^ = *E*_0_^+(NE)^ − *E*_0_^0^ (vertical ionisation potential in the NE state); VIP^EQ^ = *E*_0_^+(EQ)^ − *E*_0_^0^ (vertical ionisation potential in the EQ state); AIP = *E*_+_^+^ − *E*_0_^0^ (adiabatic ionisation potential in the relaxed solvent); VEA^NE^ = *E*_0_^−(NE)^ − *E*_0_^0^ (vertical electron affinity in the NE state); VEA^EQ^ = *E*_0_^−(EQ)^ − *E*_0_^0^ (vertical electron affinity in the EQ state); AEA = *E*_0_^0^ − *E*_−_^−^ (adiabatic electron affinity).

All the above theoretical calculations were performed using the Gaussian G16 (version C.01) software package [49].

## 5. Conclusions

A delicate redox homeostasis is crucial for intracellular signalling ROS-dependent pathways. An overload or reduction in their levels has physiological consequences, which cause pathological outcomes [4].

The activity of various harmful factors can lead to different cell macromolecules becoming damaged such as the cytoplasm membrane, proteins, lipids, and nucleic acids, which if left unrepaired, give rise to cell dysfunction, physiological aberrations, mutations, and/or lead to ageing and cancerogenesis. In this study, the ionisation potentials of low-weight antioxidants compared with guanine derivatives were considered. The obtained results showed the following order of the adiabatic ionisation potential: NAC > Vit C > Melatonin > Caffeine >Uric Acid and the adiabatic electron affinity: Vit C > Uric Acid > NAC > Caffeine > Melatonin. It should be highlighted that the purine derivatives Gua, ^OXO^Gua, Guo, dGuo, ^OXO^Guo, and ^OXO^dGuo exhibit a lower adiabatic potential than the discussed antioxidants. Therefore, their presence in the cytoplasm can protect the intracellular molecules from RO/NS and other highly reactive molecules such as radicals and aldehydes.

A comparative analysis of the obtained data indicates that Uric Acid, a common antioxidant in plasma, has the lowest AIP value with almost the highest electron affinity. The above, together with its lowest chemical hardness and highest chemical softness in adiabatic radical anion and cation states, makes it a particularly valuable antioxidant. Its presence in extracellular fluids can protect the cytoplasm membrane from the uptake of reactive species associated with diet, cigarette smoke, or ionisation radiation.

The mutual communication between proteins, lipids, and nucleotides is vital in cell machinery and is of high scientific value. Investigating this communication process could enhance our understanding of the acceleration of harmful or undesirable processes like carcinogenesis, ageing, and signal transduction. Additionally, it could bring about the improved effectiveness and safety of anticancer radiotherapy/chemotherapy or combined therapies, with a subsequent increase in the quality of life for patients undergoing treatment for cancer.

## 6. Limitations and Further Remarks

The results presented above indicate that low-molecular-weight antioxidants ingested with food can play a significant role in redox processes in extracellular and intracellular fluids, effectively protecting macromolecules from the effects of reactive oxygen/nitrogen species. However, an overload of such antioxidants can interfere with charge transfer, ultimately having an impact on such processes as DNA lesion recognition and repair. With this in mind, investigating the ionisation potential and electron affinity of various active molecules present in food would seem to be justified. Because of the labour-intensive nature and difficulties associated with experimental IP and AE measurement in the aqueous phase, using theoretical methods would appear to be a promising alternative. The current increases in computational power allow the application of a high level of theory, e.g., CCSD(T) with a complete basis set for the computation of the parameters above [50,51]. This, together with geometry optimisation at the same theoretical level yields results comparable with the experimental data. Unfortunately, at present, the application of the above is limited to small molecules or the energy calculation of medium-sized systems. It should be highlighted that different theoretical methods applied to the same problem can lead to different results. The above depends not only on the used methodology, e.g., DFT, MP, and CC but also depends on the used basis set. Therefore, the deep validation and benchmark process are highly demanded; for more details please see the valuable review articles [52,53,54]. The human body consists of around 36 trillion cells that are mutually interacting. In many cases, the information is obscured, which hampers their formulation with the clarity needed for theoretical calculations. The rise in the use of theoretical chemistry methodology and its increasing accuracy as a major rationalising tool for biology is commonly accepted. Additionally, theoretically predicting the ability of molecules to deactivate free radicals or scavenge electrons is important in our quest to colonize worlds beyond Earth. Food stability, safety, and the protection of human body cells from ionisation radiation (cosmic) all require a study of astronaut safety protocols and research into nutrition, among other things. On the other hand, as life expectancy increases, so too do incidents of cancer. The slowdown and fallibility of DNA damage repair machinery is also an age-dependent process. It is thus vital to consider and further investigate “patient-friendly” radiotherapy treatments that use ionisation radiation to kill cancer cells. The antioxidant pool could be crucial for effective treatment, which can be predicted by theoretical studies as presented here. New forays into radiotherapy are necessary to make the dose delivery more effective while reducing the detrimental and highly undesirable effects on healthy tissue surrounding a tumour. Yet an overload of antioxidants can reduce or even completely suppress any therapeutic effect. As presented in the article, the influence of small molecules on charge transfer and its subsequent effect on DNA damage recognition by repair proteins warrants future systematic theoretical investigation at the high level of theory as CCSD(T) in the condensed, i.e., aqueous phase.

## Figures and Tables

**Table 1:** The electronic properties, in [eV], of selected antioxidants and guanine derivatives: the vertical/adiabatic ionisation potential (VIP/AIP) and the vertical (VEA) and adiabatic (AEA) electron affinity as well as energies of highest occupied and lowest unoccupied molecular orbitals (HOMO, LUMO) calculated at the MP-2/aug-cc-pVTZ level of theory in the aqueous phase. Additionally, the energetic gap between HOMO and LUMO was focused as *ΔE^H-L^* in eV. The raw data orbitals have been given in the Supplementary Materials (Table S1). The graphical representation of valence orbitals and spin distribution are focused on in Figure S1 in the Supplementary Materials.

Compound	HOMO	LUMO	Δ*E^H-L^*	VIP^NE^	VIP^EQ^	AIP	VEA^NE^	VEA^EQ^	AEA
**Antioxidants**									
Caffeine	−8.64	0.78	7.86	7.96	6.56	6.56	−0.06	0.76	1.10
Uric Acid	−9.11	0.80	8.31	7.68	6.61	6.21	−0.08	0.85	1.58
NAC	−10.13	0.82	9.30	8.58	7.16	7.04	−0.10	0.51	1.46
Vit C	−9.71	0.79	8.92	8.50	7.31	6.82	−0.04	0.88	1.66
Melatonin	−8.38	0.94	6.77	7.23	6.62	6.57	−0.16	0.49	0.68
**Guanine derivatives**									
^OXO^Gua	−8.22	0.98	7.25	7.48	6.44	5.94	−0.08	0.56	1.14
^OXO^dGuo	−8.37	0.94	7.43	7.46	6.47	5.92	−0.12	0.61	1.16
^OXO^Guo	−8.40	0.93	7.46	7.47	6.50	5.95	−0.11	0.64	1.36
Gua	−8.31	0.77	7.53	8.03	6.95	6.42	−0.09	0.31	0.92
dGuo	−8.38	0.94	7.44	7.96	6.95	6.37	−0.11	0.17	0.89
Guo	−8.38	0.94	7.45	7.97	6.96	6.38	−0.17	0.16	0.88

**Table 2:** The reactivity descriptors of selected antioxidants and guanine derivatives (canonical and oxidised) calculated at the MP-2/aug-cc-pVTZ level of theory in the aqueous phase and given in eV. The valence molecular orbital energy has been used according to Koopmans’ theorem. The energy differences between VIP and VEA were calculated in non-equilibrated/equilibrated solvent–solute interactions, i.e., Vertical^NE^ and Vertical^EQ^, respectively. The parameters obtained after radical anion and cation relaxation are noted as adiabatic.

Comp.	Mode/State	Parameters [eV]				Comp.	Mode/State	Parameters [eV]			
		*η*	*S*	*μ*	*ω*			*η*	*S*	*μ*	*ω*
Caffeine	Koopmans	3.93	0.25	−4.71	2.82	Gua	Koopmans	3.77	0.27	−4.54	2.74
	Vertical ^NE^	4.01	0.25	−3.95	1.95		Vertical ^NE^	4.06	0.25	−3.97	1.94
	Vertical ^EQ^	2.90	0.34	−3.66	2.31		Vertical ^EQ^	3.32	0.30	−3.63	1.98
	Adiabatic	2.73	0.37	−3.83	2.69		Adiabatic	2.75	0.36	−3.67	2.45
Uric Acid	Koopmans	4.16	0.24	−4.95	2.95	^OXO^Gua	Koopmans	3.62	0.28	−4.60	2.92
	Vertical ^NE^	3.88	0.26	−3.80	1.86		Vertical ^NE^	3.78	0.26	−3.70	1.81
	Vertical ^EQ^	2.88	0.35	−3.73	2.42		Vertical ^EQ^	2.94	0.34	−3.50	2.08
	Adiabatic	2.32	0.43	−3.90	3.28		Adiabatic	2.40	0.42	−3.54	2.61
NAC	Koopmans	4.65	0.21	−5.47	3.22	^OXO^dGuo	Koopmans	3.72	0.27	−4.66	2.92
	Vertical ^NE^	4.34	0.23	−4.24	2.07		Vertical ^NE^	3.79	0.26	−3.67	1.78
	Vertical ^EQ^	3.33	0.30	−3.84	2.21		Vertical ^EQ^	2.93	0.34	−3.54	2.14
	Adiabatic	2.79	0.36	−4.25	3.24		Adiabatic	2.38	0.42	−3.54	2.63
Vit C	Koopmans	4.46	0.22	−5.25	3.09	^OXO^Guo	Koopmans	3.73	0.27	−4.67	2.92
	Vertical ^NE^	4.27	0.23	−4.23	2.10		Vertical ^NE^	3.79	0.26	−3.68	1.79
	Vertical ^EQ^	3.22	0.31	−4.10	2.61		Vertical ^EQ^	2.93	0.34	−3.54	2.14
	Adiabatic	2.58	0.39	−4.24	3.48		Adiabatic	2.93	0.34	−3.57	2.17
Melatonin	Koopmans	3.39	0.30	−4.33	2.78	dGuo	Koopmans	3.72	0.27	−4.66	2.92
	Vertical ^NE^	3.70	0.27	−3.53	1.69		Vertical ^NE^	4.04	0.25	−3.93	1.91
	Vertical ^EQ^	3.07	0.33	−3.56	2.06		Vertical ^EQ^	3.39	0.29	−3.56	1.87
	Adiabatic	2.76	0.36	−3.44	2.14		Adiabatic	2.74	0.36	−3.63	2.40
						Guo	Koopmans	3.72	0.27	−4.66	2.92
							Vertical NE	4.07	0.25	−3.90	1.87
							Vertical EQ	3.40	0.29	−3.56	1.86
							Adiabatic	2.75	0.36	−3.63	2.40

**Table 3:** The Hirshfeld spin distribution (only atoms with highest concentrations were focused on) of selected antioxidants and Gua derivatives calculated at the MP-2/aug-cc-pVTZ level of theory in the aqueous phase and given in [au × 10^−2^]. VC—vertical cation, VA—vertical anion, AC—adiabatic cation, AA—adiabatic anion, NE—non-equilibrated, EQ—equilibrated solvent-solute interaction mode. (The graphical representation of spin distribution is shown in Figure S1).

Compound	VC^NE^	VC^EQ^	AC	VA^NE^	VA^EQ^	AA
Caffeine	C5(44), O6(27)	C5(38), C8(21)	C5(38), C8(21)	C3(26), C3(20)	C8(37), C6(31)	C8(63), N7(13)
Uric Acid	C5(40), O6(26)	C5(40), O6(27)	O6(47), C5(36)	N1(35), N3(31)	C4(40), C6(37)	C6(18), C5(50)
NAC	S9(95)	S9(95)	S9(93)	C5(24), S9(23)	C1(57), O1(21)	C1(63), O1(20)
Vit C	C3(34), C4(25)	C3(34), C4(27)	C3(33), C4(26)	O4(40), C7(21)	C2(41), C3(35)	C2(41), C3(35)
Melatonin	C3(37), N1(36)	C3(39), N1(44)	C3(38), N1(44)	C2(12), C7(9)	C2(32), C7(31)	C7(37), C4(31)
^OXO^Gua	C5(41), O6(34)	C5(41), O6(35)	C5(67), C6(25)	N2(62), N1(16)	C4(40), C6(34)	C4(54), C6(20)
^OXO^dGuo	C5(41), C6(36)	C5(41), O6(37)	C5(44), O6(36)	N2(53), N(20)	C4(41), C6(34)	C4(55), C6(21)
^OXO^rGuo	C5(41), O6(36)	C5(41), O6(37)	C5(42), O6(21)	N2(55), N1(19)	C4(41), C6(35)	C4(54), C6(19)
Gua	C5(40), C8(31)	C5(41), C8(32)	C5(34), C8(31)	N9(27), N2(26)	C4(41), C6(33)	C6(58), O6(19)
dGuo	C5(40), C8(32)	C5(41), C8(32)	C5(33), C8(30)	N2(63), N1(13)	C6(41), C4(36)	C6(57), O6(18)
rGuo	C5(41), C8(32)	C5(41), C8(31)	C5(41), C8(30)	N2(64), N1(15)	C6(40), C4(36)	C6(59), O6(18)

## Data Availability

Data are contained within the article and Supplementary Materials.

## Supplementary Materials

The following supporting information can be downloaded at https://www.mdpi.com/article/10.3390/molecules29245944/s1, Table S1: The electronic state energy in Hartree and dipole moment (DM) in Debye calculated at MP-2/aug-cc-pVTZ level of theory in the condensed phase using a non-equilibrated and equilibrated solvent–solute interaction. Figure S1. Graphical visualisation of HOMO, LUMO, and spin distribution calculated at the MP-2/aug-cc-pVTZ level of theory in the aqueous phase of the molecules discussed in the article in their adiabatic and vertical radical cation and anion forms. The non-equilibrated (NE) and equilibrated (EQ) solvent–solute interaction have been taken into consideration. The optimised structures of the discussed compounds in neutral, cationic, and anionic forms, such as those in *.pdb files, were compressed into a zipped PDB_structure file.
