# Supplementary material for: A Comparison of the Electronic Properties of Selected Antioxidants Vitamin C, Uric Acid, NAC and Melatonin with Guanosine Derivatives: A Theoretical Study
Source: Molecules. 2024 Dec 17;29(24):5944. doi: 10.3390/molecules29245944 (PMC11678427; doi:10.3390/molecules29245944)
Supplement: Supplementary file 1 [file molecules-29-05944-s001.zip › Supplementary Materials.pdf]

Supplementary Materials

A comparison of the electronic properties of selected antioxidants Vitamin C, Uric Acid, NAC and Melatonin with guanosine derivatives. A theoretical study.

Boleslaw T. Karwowski

Table S1. The Electronic state Energy in Hartree and Dipole Moment (DM) in Debye calculated on MP-2/aug-cc-pVTZ level of theory in condensed phase using the non-equilibrated and equilibrated solvent solute interaction.

		Electronic state Energy in Hartree and Dipole Moment (DM) in Debay						
**Compound**		**Adiabatic Cation**	**Vertical Cation Equilibrated**	**Vertical Cation NON-Equilibrated**	**Neutral**	**Vertical Anion NON-Equilibrated**	**Vertical Anion Equilibrated**	**Adiabatic Anion**
**Caffeine**	Energy	-678.94585	-678.945849	-678.894434	-679.186937	-679.184606	-679.21	-679.227291
	DM	7.34	7.34	8.40	6.07	5.66	4.03	2.87
**Uric Acid**	Energy	-636.447111	-636.432661	-636.393381	-636.675454	-636.672552	-636.706637	-636.733522
	DM	5.13	4.49	4.56	4.96	10.85	7.49	5.53
**Guo**	Energy	-541.417133	-541.397743	-541.357959	-541.652997	-541.649524	-541.6642155	-541.686666
	DM	9.48	7.87	8.27	9.68	5.72	11.94	13.21
**NAC**	Energy	-873.080418	-873.075808	-873.023672	-873.339115	-873.335357	-873.357984	-873.392655
	DM	17.09	16.68	15.22	5.60	1.97	9.06	9.57
**Vit C**	Energy	-683.475984	-683.458161	-683.414446	-683.726774	-683.725364	-683.759126	-683.787946
	DM	12.96	12.32	12.18	12.73	8.63	18.26	14.68
**^{oxo}Guo**	Energy	-616.588927	-616.570613	-616.532094	-616.807153	-616.804379	-616.827882	-616.849
	DM	8.79	8.55	8.79	9.90	11.09	8.93	7.44
**Gua**	Energy	-1035.987315	-1035.966083	-1035.928874	-1036.221739	-1036.215604	-1036.227778	-1036.25418
	DM	10.16	8.44	7.99	8.42	8.19	19.77	20.36
**^{oxo}Gua**	Energy	-1111.099412	-1111.079258	-1111.043372	-1111.317968	-1111.313859	-1111.341458	-1111.36793
	DM	14.13	14.54	13.66	9.20	16.20	11.73	10.02
**dGua**	Energy	-960.916642	-960.895593	-960.8584	-961.150911	-961.146728	-961.157053	-961.183713
	DM	13.60	11.81	11.54	10.65	22.99	19.18	20.72
**^{oxo}dGua**	Energy	-1036.034092	-1036.013762	-1035.977552	-1036.251558	-1036.24717	-1036.273861	-1036.294133
	DM	11.20	11.51	10.67	7.83	17.95	12.60	11.21

Figure S1. Graphical visualization of HOMO, LUMO and spin distribution calculated at the MP-2/aug-cc-pVTZ level of theory in the aqueous phase of the molecules discussed in the article in their Adiabatic and Vertical radical cation and anion forms. The non-equilibrated (NE) and equilibrated (EQ) solvent-solute interaction has been take into consideration.

**Caffeine**			
	**HOMO**	**LUMO**	**SPIN**
**Vertical NE Anion**	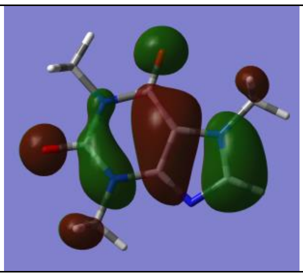	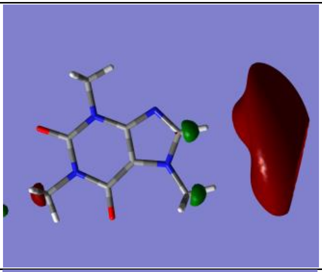	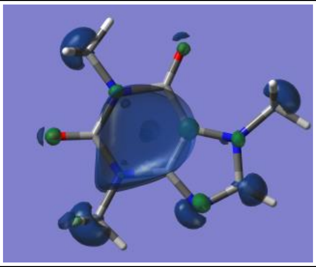
**Vertical EQ Anion**	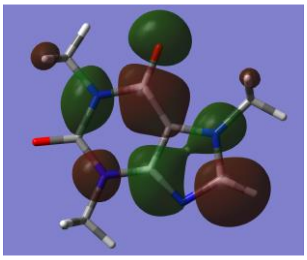	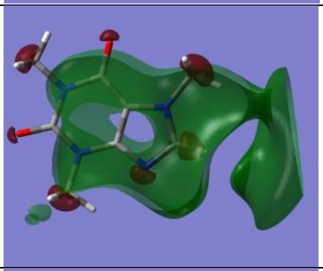	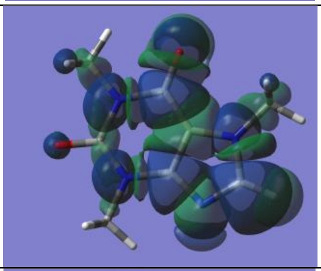
**Adiabatic Anion**	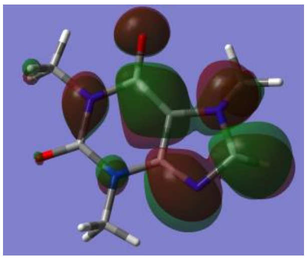	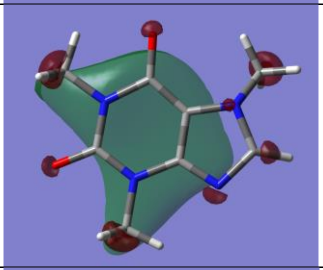	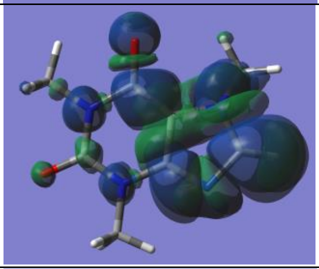
**Vertical NE Cation**	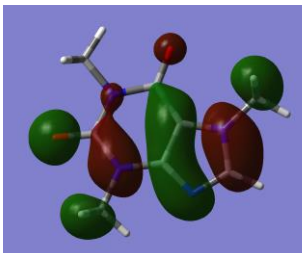	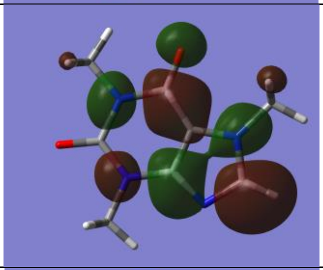	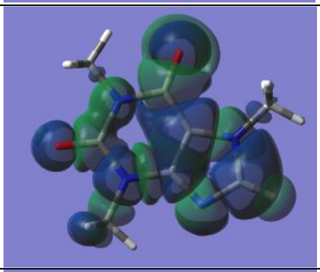
**Vertical EQ Cation**	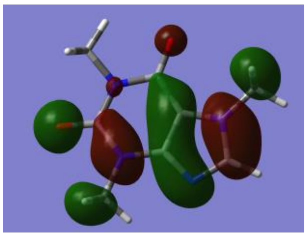	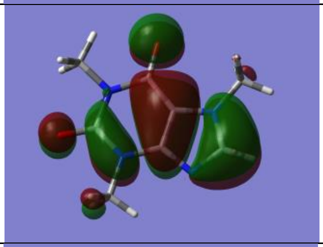	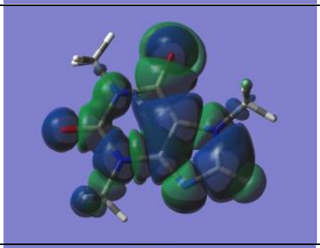
**Adiabatic Cation**	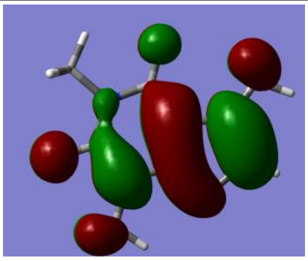	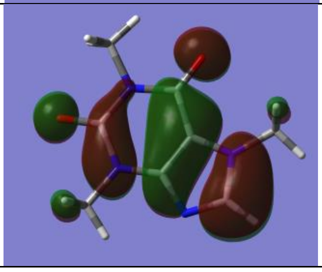	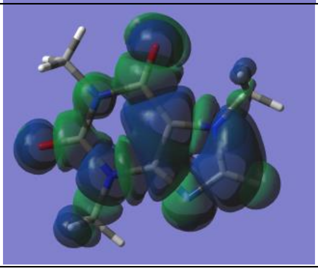

Figure S1. cont.

Melatonin			
	**HOMO**	**LUMO**	**SPIN**
**Vertical NE Anion**	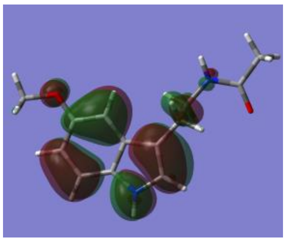	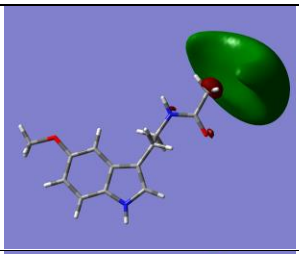	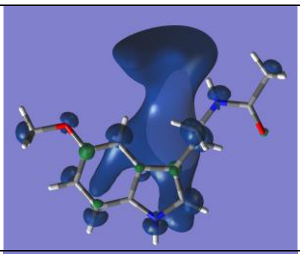
**Vertical EQ Anion**	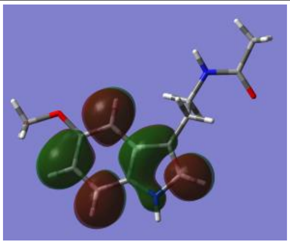	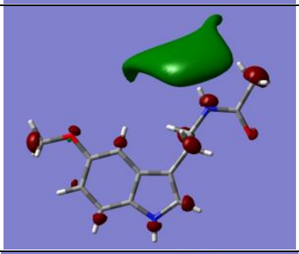	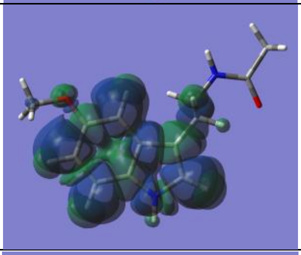
**Adiabatic Anion**	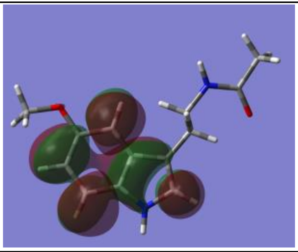	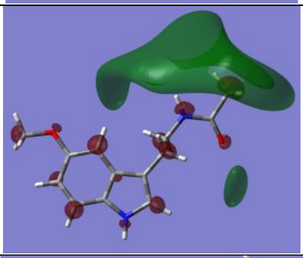	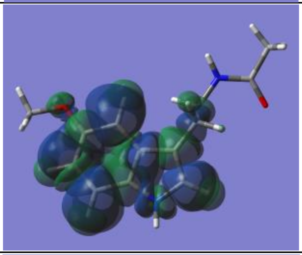
**Vertical NE Cation**	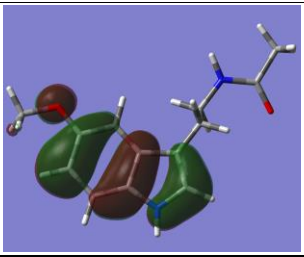	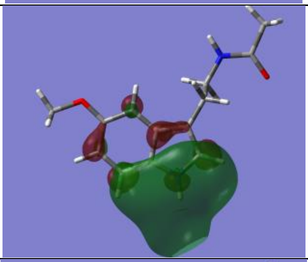	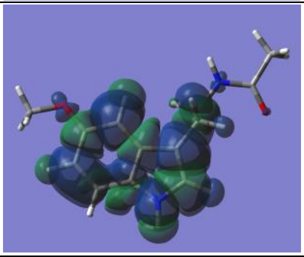
**Vertical EQ Cation**	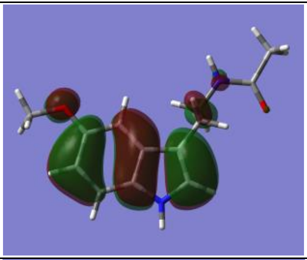	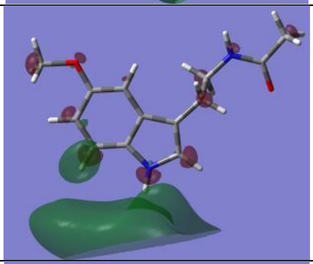	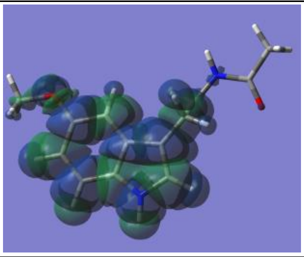
**Adiabatic Cation**	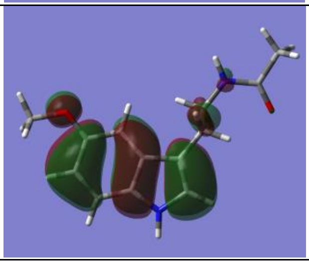	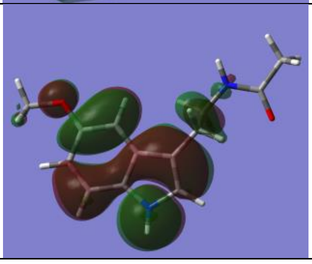	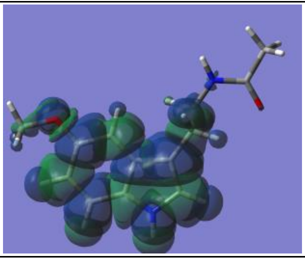

Figure S1. cont.

Uric acid			
	**HOMO**	**LUMO**	**SPIN**
**Vertical NE Anion**	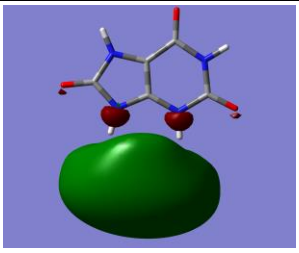	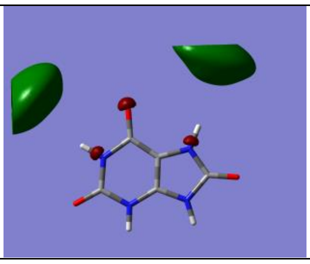	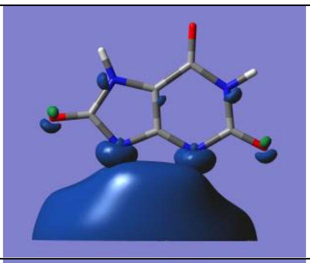
**Vertical EQ Anion**	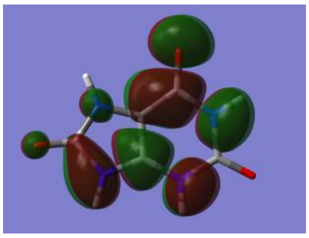	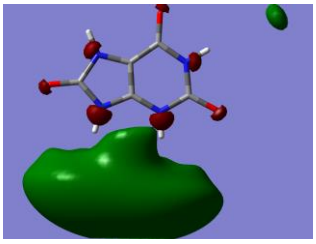	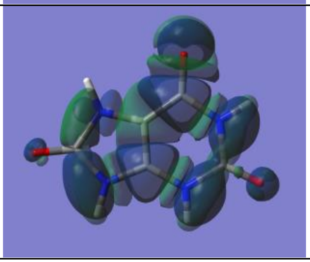
**Adiabatic Anion**	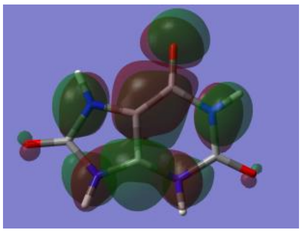	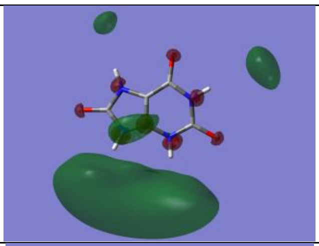	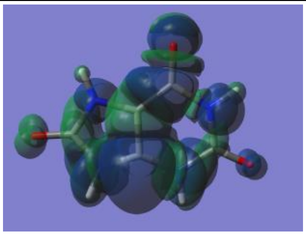
**Vertical NE Cation**	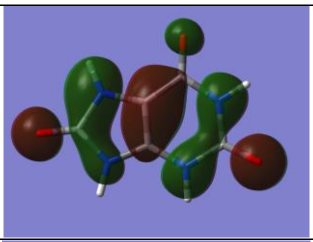	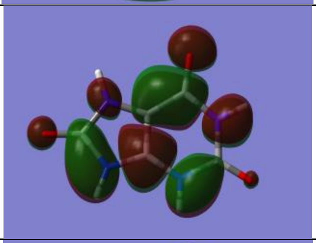	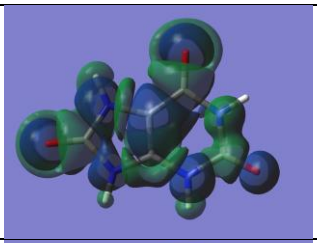
**Vertical EQ Cation**	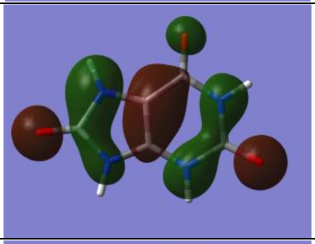	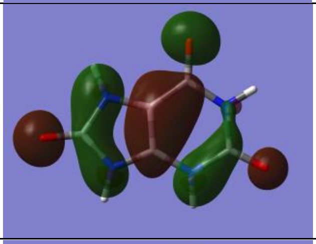	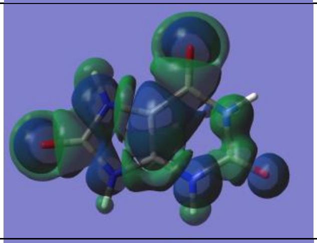
**Adiabatic Cation**	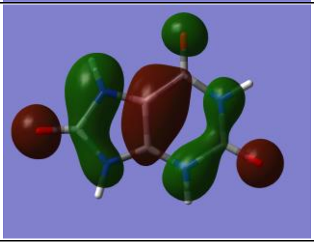	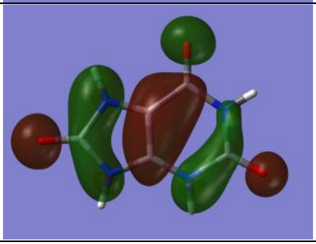	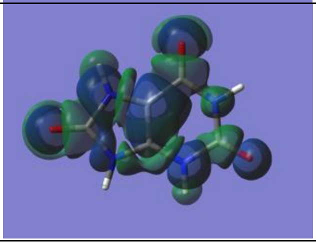

Figure S1. cont.

Vit C			
	HOMO	LUMO	SPIN
**Vertical NE Anion**	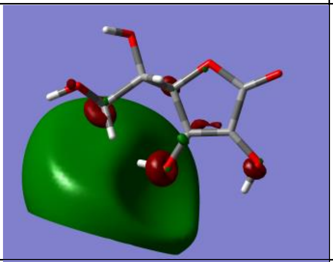	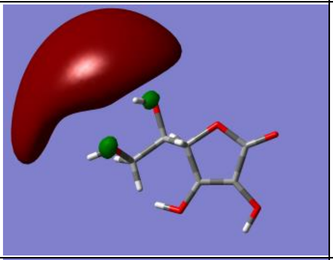	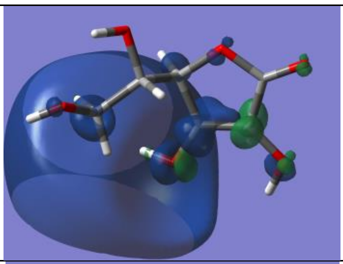
**Vertical EQ Anion**	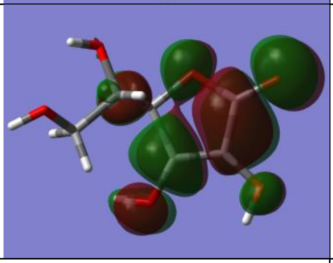	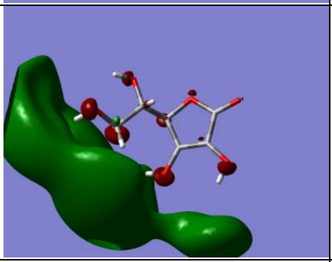	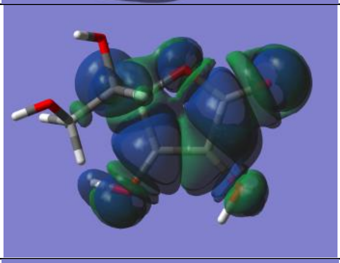
**Adiabatic Anion**	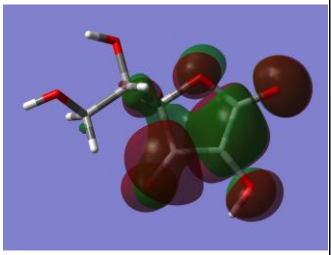	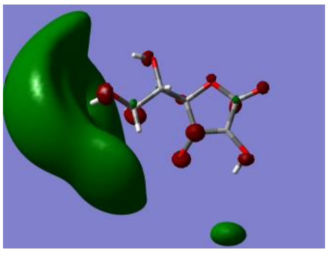	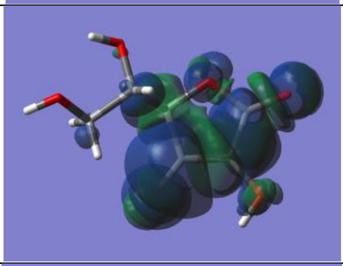
**Vertical NE Cation**	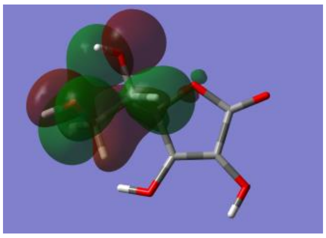	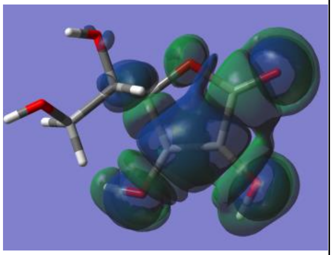	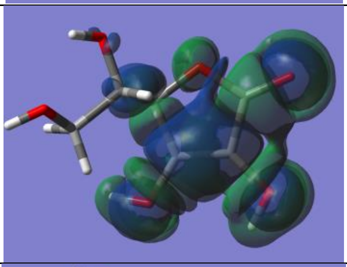
**Vertical EQ Cation**	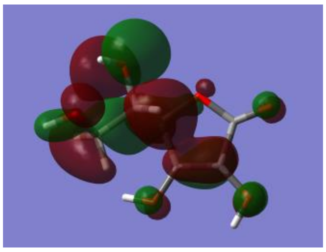	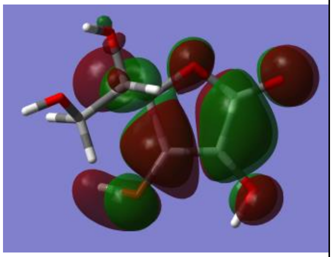	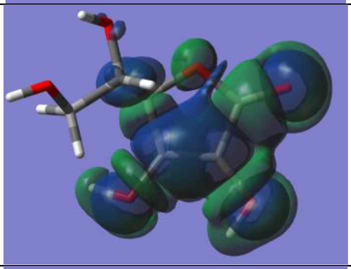
**Adiabatic Cation**	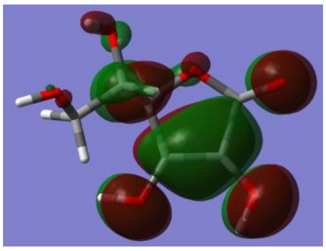	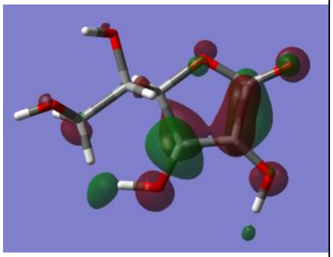	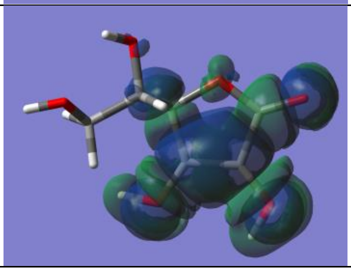

Figure S1. cont.

N-acethyl-cysteine (NAC)			
	**HOMO**	**LUMO**	**SPIN**
**Vertical NE Anion**	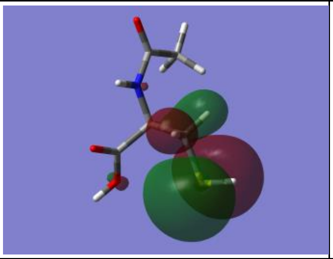	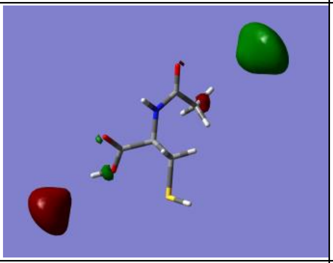	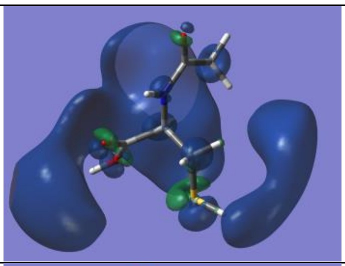
**Vertical EQ Anion**	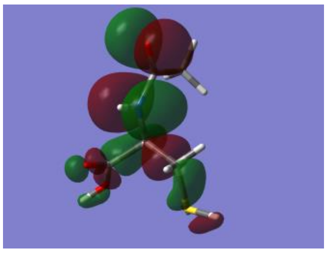	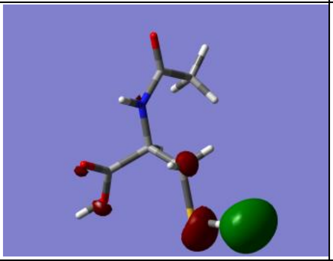	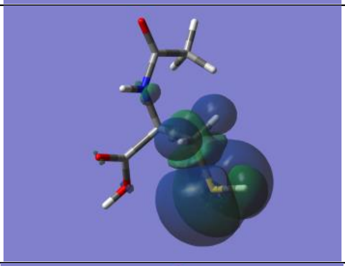
**Adiabatic Anion**	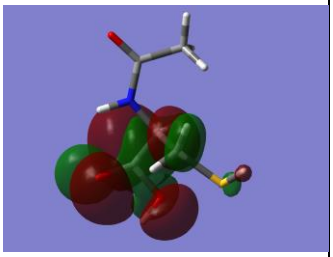	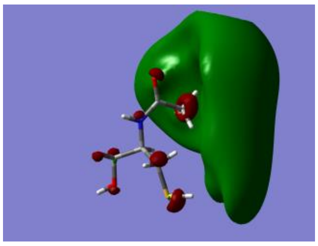	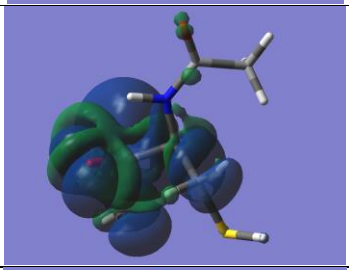
**Vertical NE Cation**	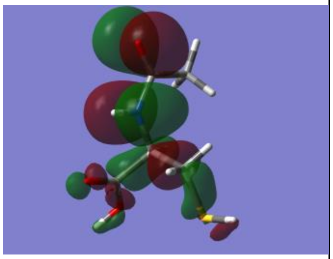	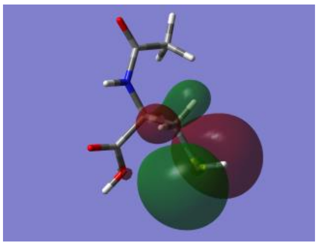	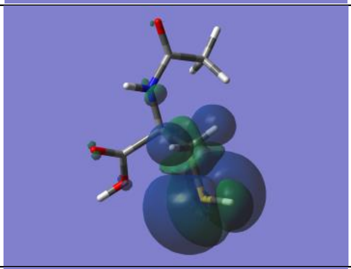
**Vertical EQ Cation**	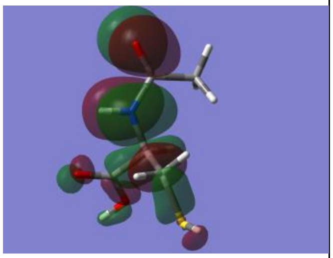	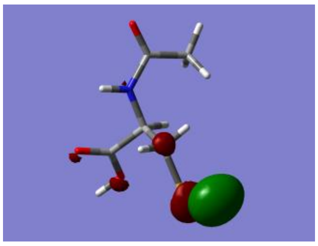	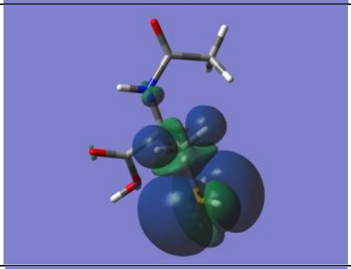
**Adiabatic Cation**	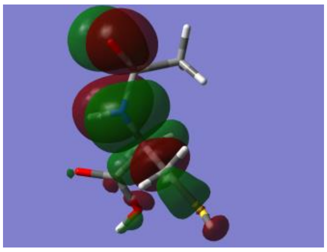	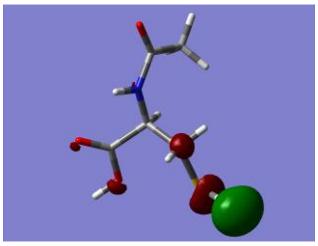	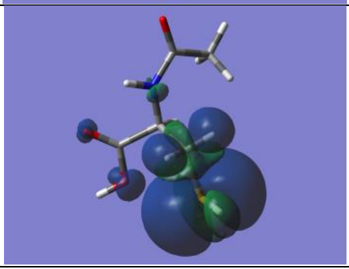

Figure S1. cont.

**Gua** (Guanine)			
	**HOMO**	**LUMO**	**SPIN**
**Vertical NE Anion**	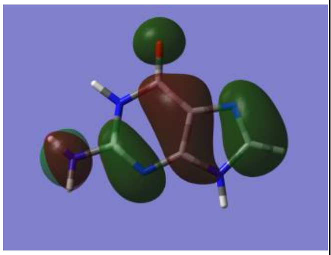	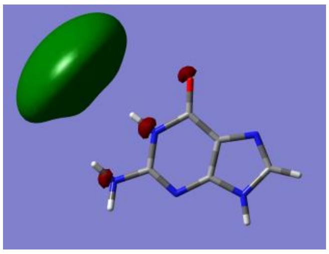	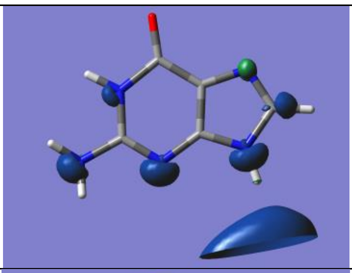
**Vertical EQ Anion**	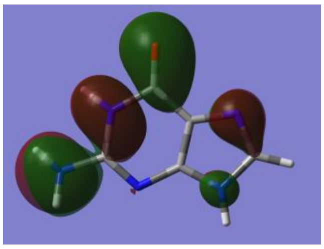	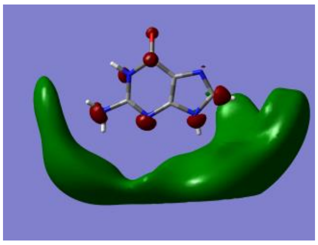	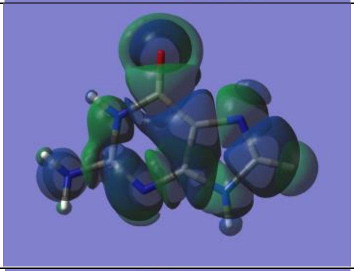
**Adiabatic Anion**	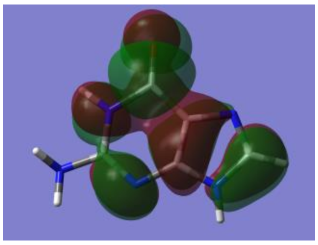	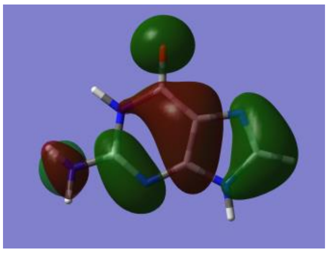	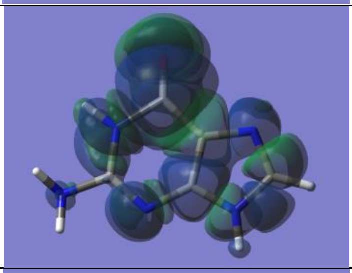
**Vertical NE Cation**	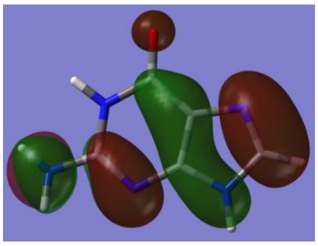	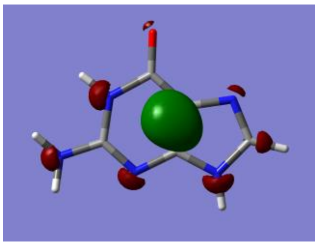	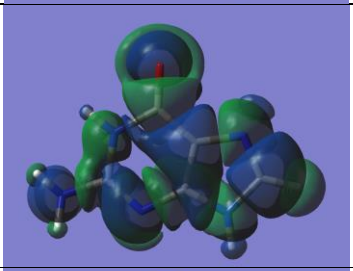
**Vertical EQ Cation**	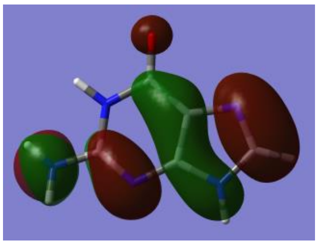	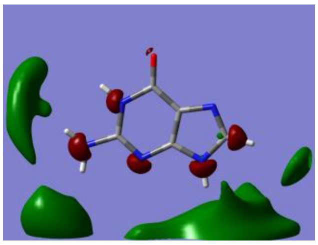	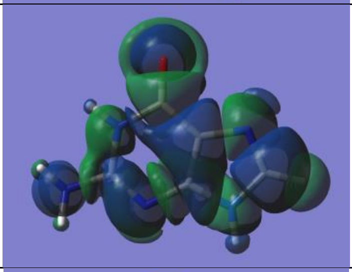
**Adiabatic Cation**	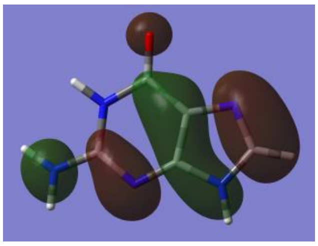	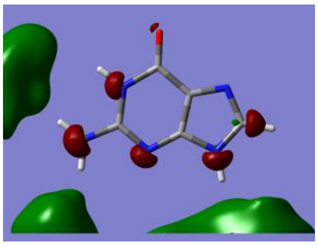	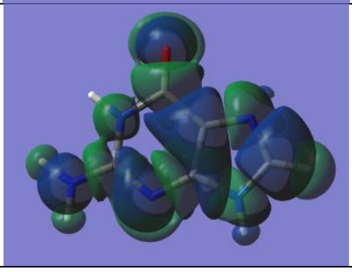

Figure S1. cont.

^{oxo}**Gua** (7,8-dihydro-8-oxo-Guanine)			
	**HOMO**	**LUMO**	**SPIN**
**Vertical NE Anion**	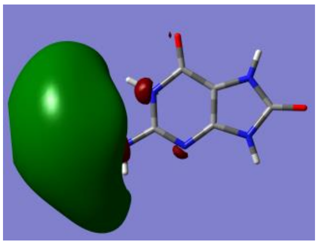	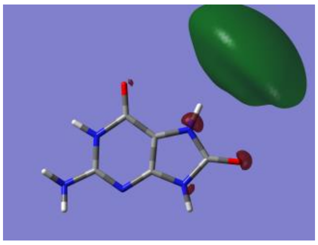	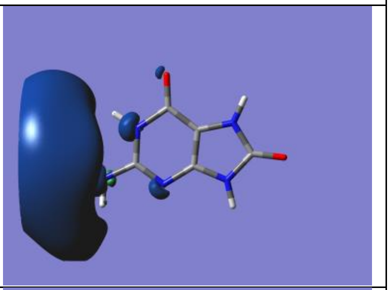
**Vertical EQ Anion**	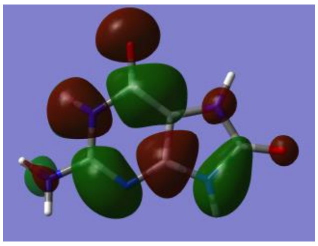	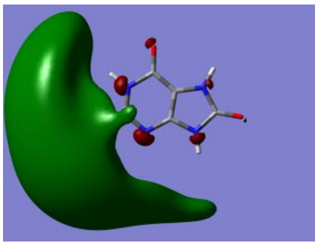	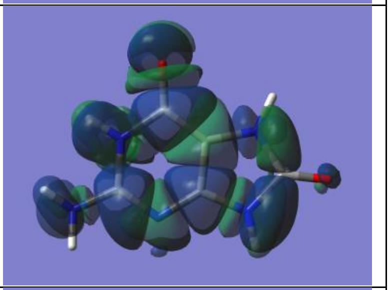
**Adiabatic Anion**	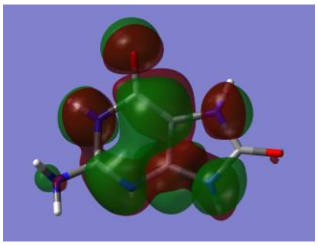	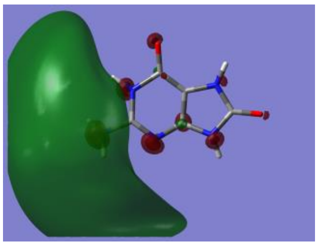	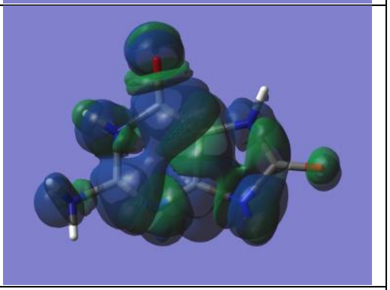
**Vertical NE Cation**	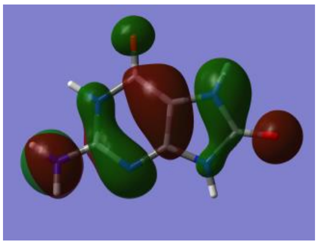	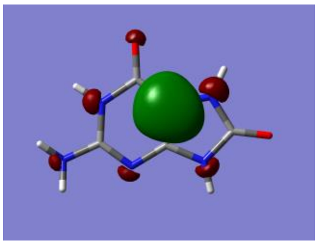	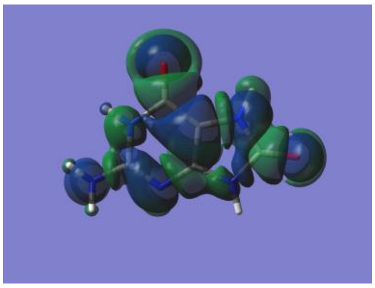
**Vertical EQ Cation**	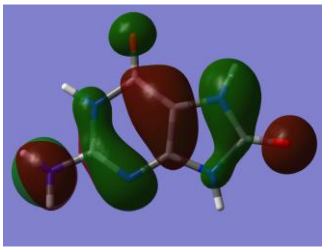	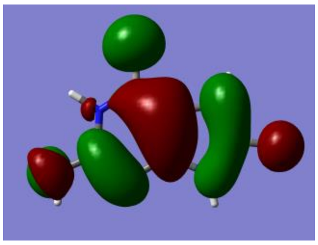	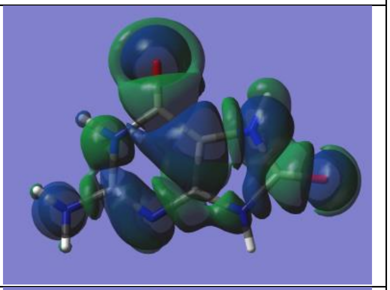
**Adiabatic Cation**	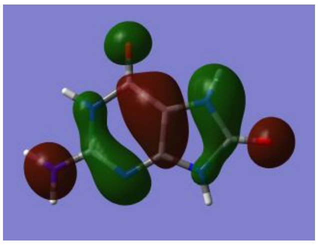	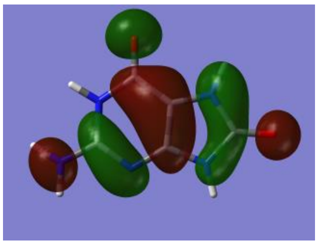	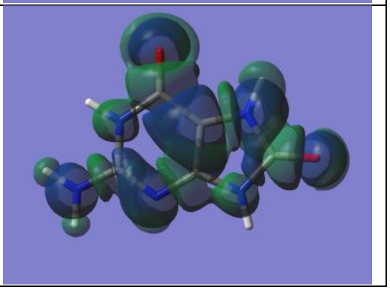

Figure S1. cont.

^{oxo}**dGuo** (7,8-dihydro-8-oxo-2-deoxyGuanosine)			
	**HOMO**	**LUMO**	**SPIN**
**Vertical NE Anion**	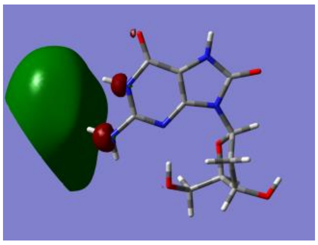	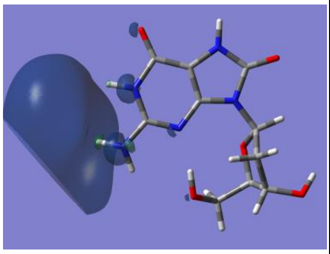	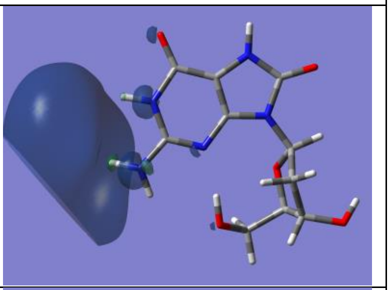
**Vertical EQ Anion**	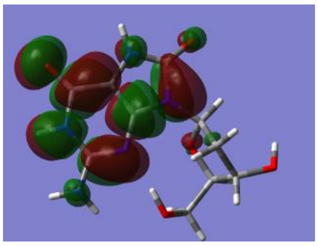	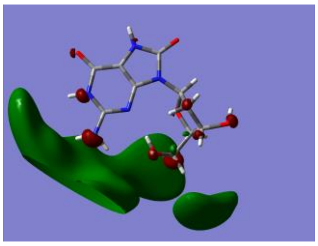	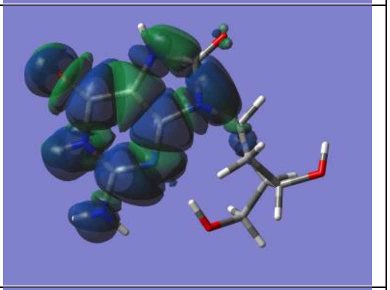
**Adiabatic Anion**	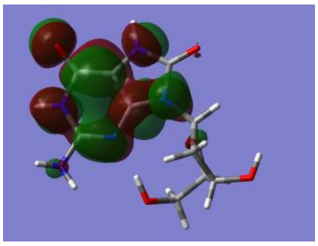	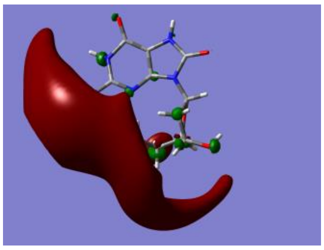	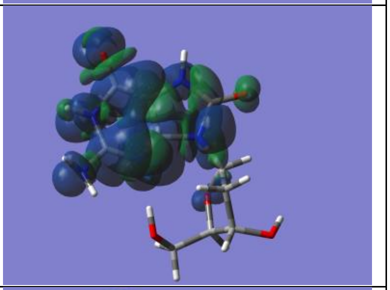
**Vertical NE Cation**	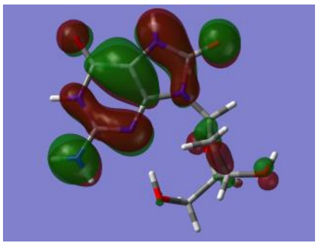	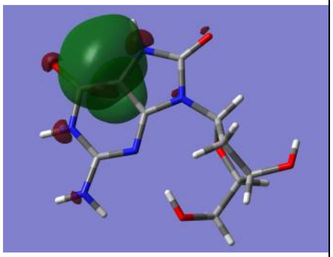	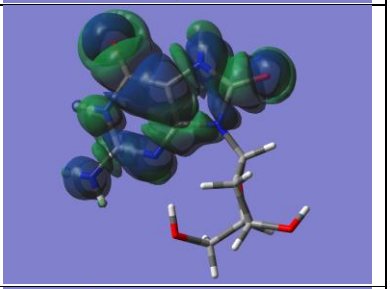
**Vertical EQ Cation**	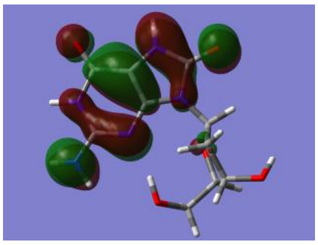	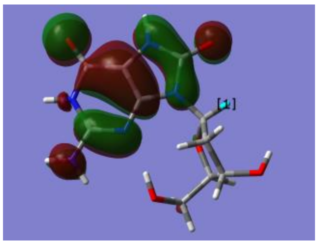	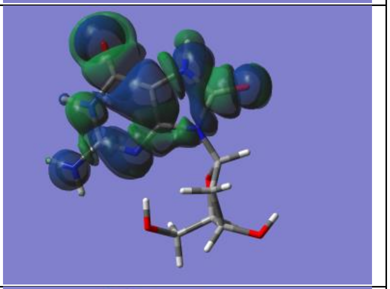
**Adiabatic Cation**	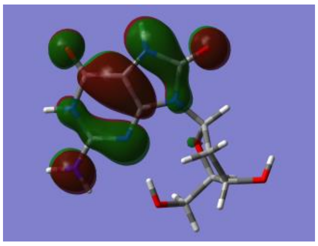	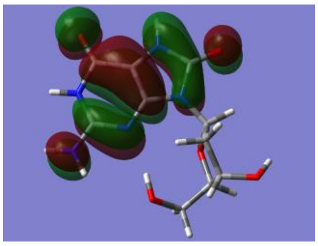	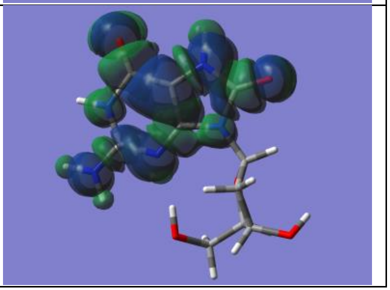

Figure S1. cont.

**dGuo** (2’-deoxyGuanosine)			
	**HOMO**	**LUMO**	**SPIN**
**Vertical NE Anion**	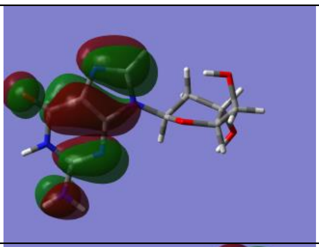	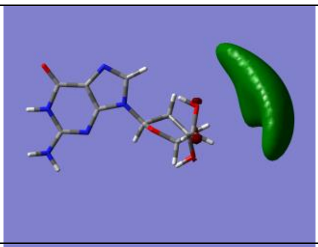	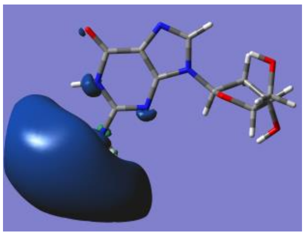
**Vertical EQ Anion**	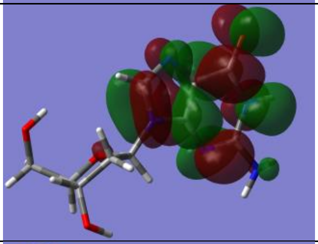	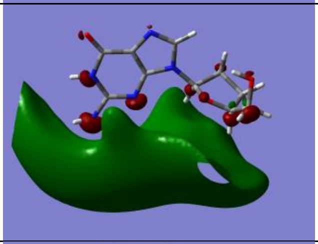	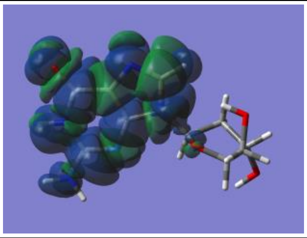
**Adiabatic Anion**	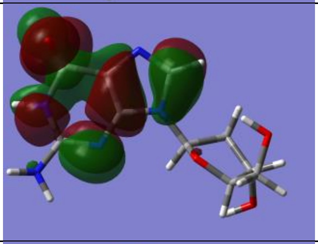	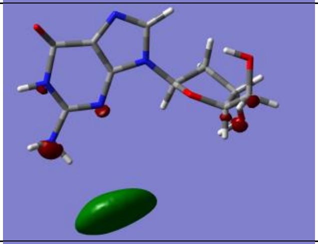	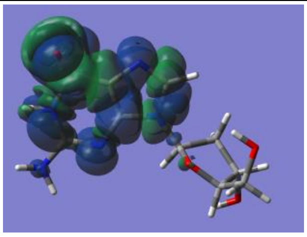
**Vertical NE Cation**	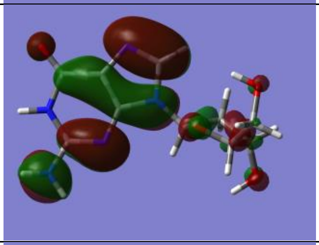	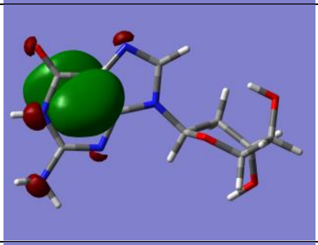	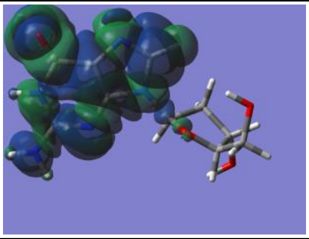
**Vertical EQ Cation**	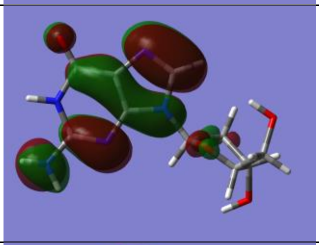	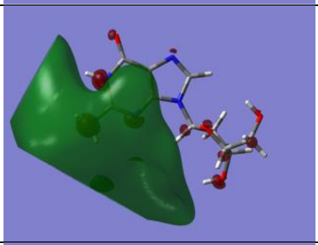	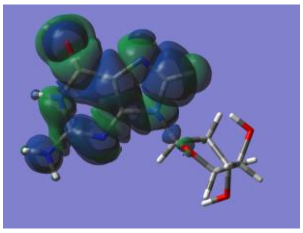
**Adiabatic Cation**	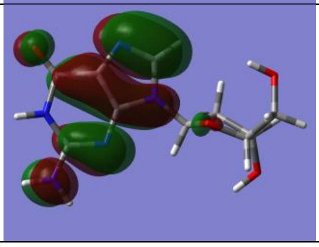	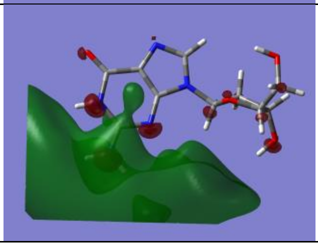	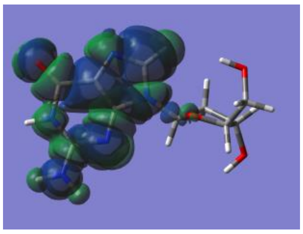

Figure S1. cont.

**Guo** (Guanosine)			
	**HOMO**	**LUMO**	**SPIN**
**Vertical NE Anion**	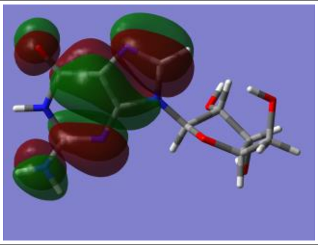	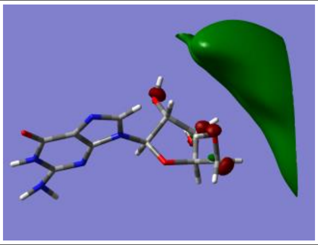	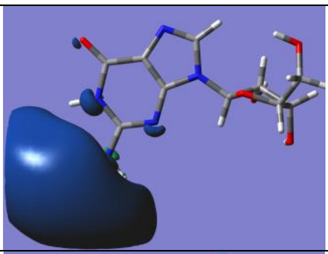
**Vertical EQ Anion**	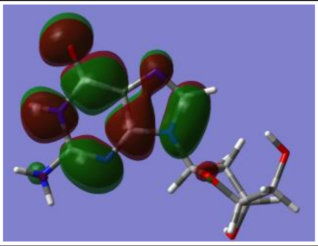	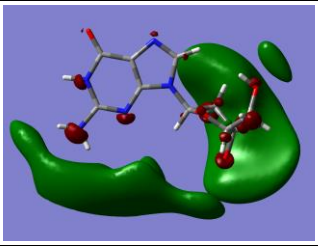	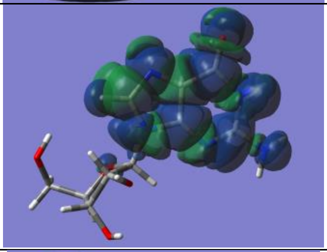
**Adiabatic Anion**	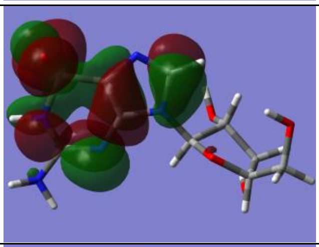	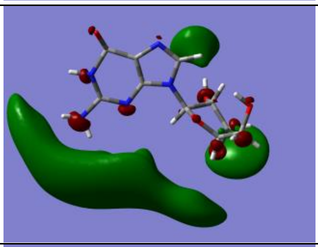	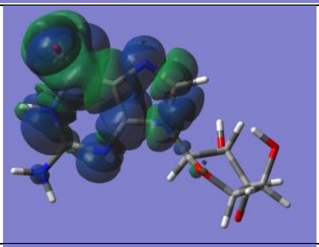
**Vertical NE Cation**	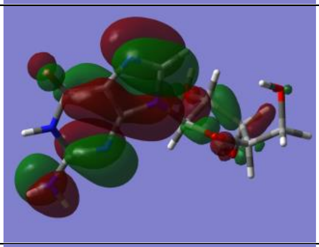	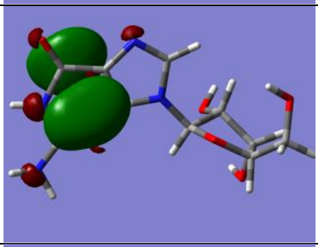	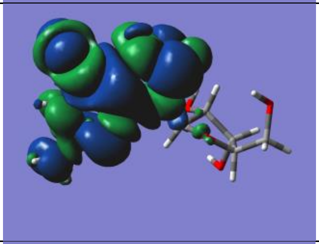
**Vertical EQ Cation**	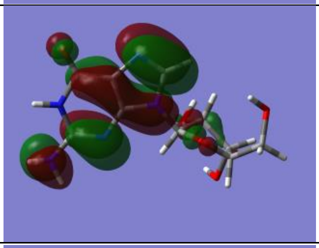	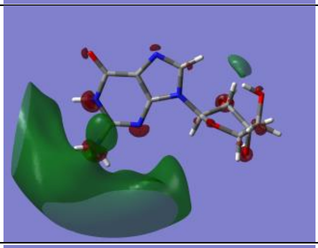	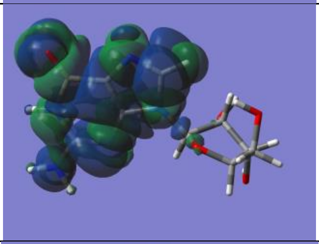
**Adiabatic Cation**	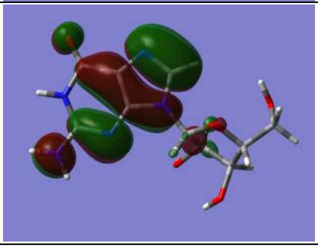	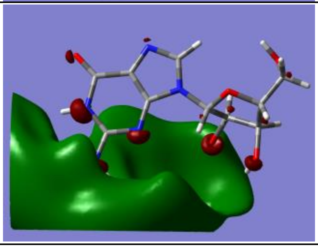	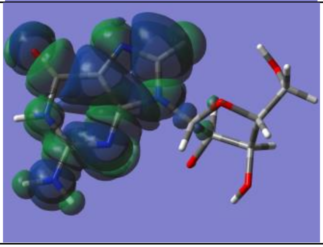

Figure S1. cont.

^{oxo}**Guo** (7,8-dihydro-8-oxo-Guanosine)			
	**HOMO**	**LUMO**	**SPIN**
**Vertical NE Anion**	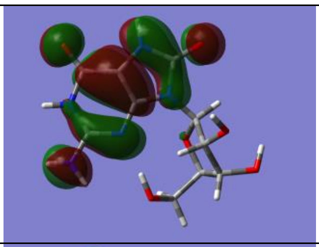	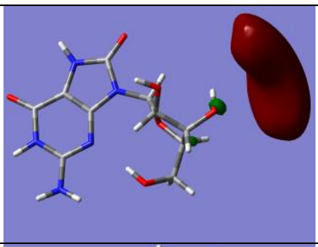	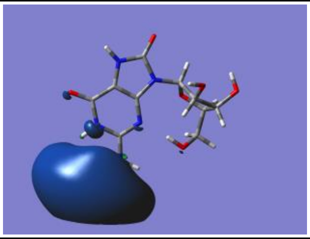
**Vertical EQ Anion**	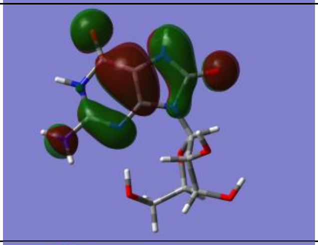	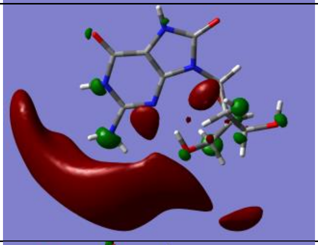	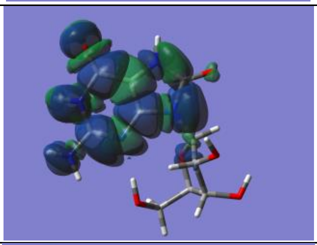
**Adiabatic Anion**	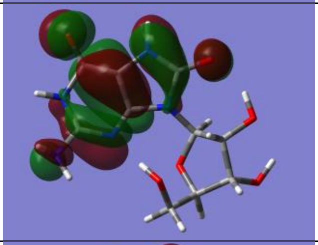	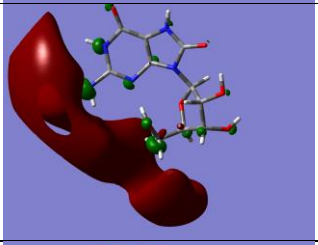	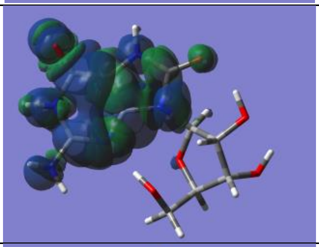
**Vertical NE Cation**	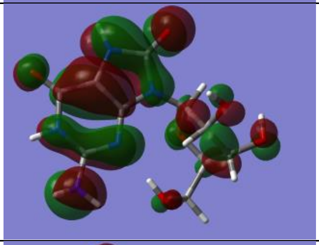	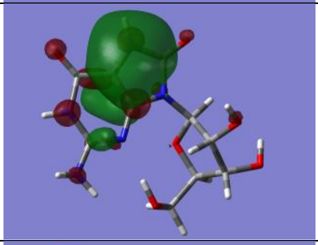	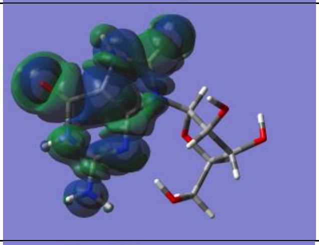
**Vertical EQ Cation**	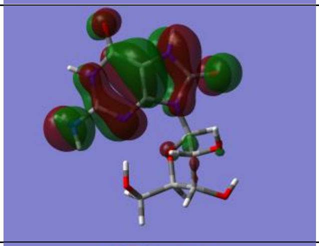	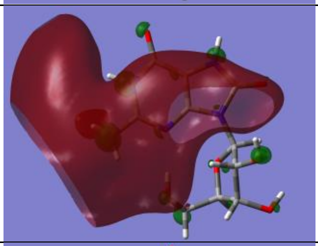	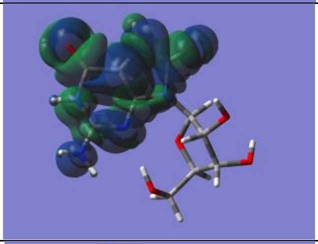
**Adiabatic Cation**	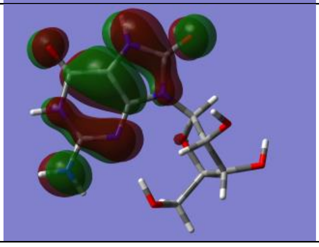	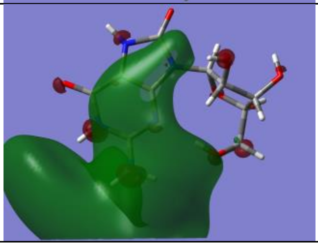	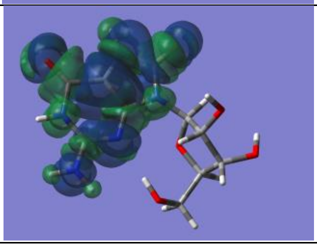
